# Patients Pay £10,000

**Published:** 1921-03-05

**Authors:** 


### LONDON HOSPITAL'S POSITION.

### Patients Pay £10,000.

The report of the House Committee for the first quarter of the year 1921 was presented at the quarterly Court of Governors on March 2, Viscount Knutsford in the chair. The report opens by pointing out that this year must prove the most critical in the hospital's history. It has survived the past year despite serious crises, but the rocks are ever in sight.

### Lord Cave's Committee.

In the evidence given before this Committee, Lord Knutsford pointed out that the dependence of hospitals entirely on charitable support has certain weaknesses in that their geographical distribution has been haphazard, there is no co-ordination of working, no co-ordinated buying and much reduplication of work. Also, the appealing for money is done in such a way that the appeals of one hospital lessen the effectiveness of the appeal of another. Hardly any hospital is free of the paralysing influence of uncertainty as to its funds; consequently, improvements in pay, accommodation, and hours of nurses are delayed, and advances in medicine and surgery tend to be looked at askance because they entail greater expense. Whilst indicating the need for some alteration, Lord Knutsford strongly urged that the control of the voluntary hospitals should not be taken out of the hands of their present managers. If helped by the Government or municipality, such help should not carry with it representation on the board of management. The Government's help might take the form of a deduction of any gifts to hospitals from a donor's income before assessment for income tax, or estate duty might be calculated after deducting from the gross amount of the estate any sums left to hospitals, and hospitals might be excused the 10 per cent. duty on legacies. This would cost the country something, but nothing like so much as if it had to keep up all the hospitals. Lord Knutsford pleaded that the hospitals should be paid on a higher scale for work done for the Government or London County Council. At present the payments made barely cover the cost of salaries necessary for the services rendered, and no consideration is given to money spent by the hospitals in buildings and equipment and administration, without which the services required could not be rendered.

### Approved Societies and Insured Patients.

The Hospital's Chairman recently met a conference of members of approved societies, and pointed out the difficulties of the voluntary hospitals and the benefits insured people are receiving from them. It is estimated that insured patients cost the London Hospital alone £79,300 in 1919, and it will be more in 1921.

### Payment by Patients.

The installation of the department in connection with the payment by in-patients can be regarded as a very real success. The financial result of the past six months' working shows receipts totalling close on £10,000, though for some weeks after the commencement of the scheme there were a large number of patients in hospital who were exempted from payment as they had been admitted prior to opening the department. This sum does not represent the actual full total of payment by in-patients to the hospital, as, in addition, there is the payment by local authorities for certain services. The greatest care has been taken not to exact full or any payment from the very poor.

### Paulin Ward.

By the unanimous wish of the committee it has been decided that the Charlotte Ward shall be henceforth known as Paulin Ward, as some recognition of the great services rendered to the hospital by its treasurer, Mr. W. T. Paulin, who has been a member of the committee since February 1895, and treasurer since 1913. Besides almost daily attendance at the hospital, he has been one of its most generous supporters.

### Nurses' Bazaar.

Preparations are in full swing for a bazaar organised by the nursing staff to relieve the hospital in its straits. It will be held on July 6 and 7, and no effort will be spared to ensure every possible success, and to relieve people of their money in as pleasing, thorough, and honest a manner as is conceivable. The committee deeply appreciate the spirit shown by the nursing staff in their untiring efforts, ungrudgingly given in "off-duty" time, to achieve success.

The treasurer, Mr. W. T. Paulin, and Mr. Serena, a member of the committee, have jointly made a gift to the nursing staff of a hard tennis court which will add greatly to their pleasure and recreation.

